# Effect of dietary oil from *Camelina sativa* on the growth performance, fillet fatty acid profile and gut microbiome of gilthead Sea bream (*Sparus aurata*)

**DOI:** 10.7717/peerj.10430

**Published:** 2020-12-09

**Authors:** David Huyben, Simona Rimoldi, Chiara Ceccotti, Daniel Montero, Monica Betancor, Federica Iannini, Genciana Terova

**Affiliations:** 1 Department of Animal Biosciences, University of Guelph, Guelph, ON, Canada; 2 Institute of Aquaculture, Faculty of Natural Sciences,, University of Stirling, Stirling, United Kingdom; 3 Department of Biotechnology and Life Sciences, University of Insubria, Varese, Italy; 4 Grupo de Investigación en Acuicultura (GIA), IU-ECOAQUA, Universidad de Las Palmas de Gran Canaria, Las Palmas, Canary Islands, Spain; 5 Institute of Aquaculture, Faculty of Natural Sciences, University of Stirling, Stirling, United Kingdom

**Keywords:** Aquaculture, Fish oil, Gut microbiota, Lipid, Metagenome, Next-generation sequencing, Omega-3, PICRUSt

## Abstract

**Background:**

In the last two decades, research has focused on testing cheaper and sustainable alternatives to fish oil (FO), such as vegetable oils (VO), in aquafeeds. However, FO cannot be entirely replaced by VOs due to their lack of omega-3 (n-3) long-chain polyunsaturated fatty acids (LC-PUFA), particularly eicosapentaenoic (EPA; 20:5n-3) and docosahexaenoic (DHA; 22:6n-3) acids. The oilseed plant, *Camelina sativa*, may have a higher potential to replace FO since it can contains up to 40% of the omega-3 precursors *α*-linolenic acid (ALA; 18:3n-3) and linoleic acid (LA; 18:2n-6).

**Methods:**

A 90-day feeding trial was conducted with 600 gilthead sea bream (*Sparus aurata*) of 32.92 ±  0.31 g mean initial weight fed three diets that replaced 20%, 40% and 60% of FO with CO and a control diet of FO. Fish were distributed into triplicate tanks per diet and with 50 fish each in a flow-through open marine system. Growth performance and fatty acid profiles of the fillet were analysed. The Illumina MiSeq platform for sequencing of 16S rRNA gene and Mothur pipeline were used to identify bacteria in the faeces, gut mucosa and diets in addition to metagenomic analysis by PICRUSt.

**Results and Conclusions:**

The feed conversion rate and specific growth rate were not affected by diet, although final weight was significantly lower for fish fed the 60% CO diet. Reduced final weight was attributed to lower levels of EPA and DHA in the CO ingredient. The lipid profile of fillets were similar between the dietary groups in regards to total saturated, monounsaturated, PUFA (n-3 and n-6), and the ratio of n-3/n-6. Levels of EPA and DHA in the fillet reflected the progressive replacement of FO by CO in the diet and the EPA was significantly lower in fish fed the 60% CO diet, while ALA was increased. Alpha and beta-diversities of gut bacteria in both the faeces and mucosa were not affected by any dietary treatment, although a few indicator bacteria, such as *Corynebacterium* and *Rhodospirillales*, were associated with the 60% CO diet. However, lower abundance of lactic acid bacteria, specifically *Lactobacillus*, in the gut of fish fed the 60% CO diet may indicate a potential negative effect on gut microbiota. PICRUSt analysis revealed similar predictive functions of bacteria in the faeces and mucosa, although a higher abundance of *Corynebacterium* in the mucosa of fish fed 60% CO diet increased the KEGG pathway of fatty acid synthesis and may act to compensate for the lack of fatty acids in the diet. In summary, this study demonstrated that up to 40% of FO can be replaced with CO without negative effects on growth performance, fillet composition and gut microbiota of gilthead sea bream.

## Introduction

In the last two decades, research has focused on testing cheaper and sustainable alternatives to fish oil (FO) in aquafeeds that do not compromise fish growth and the omega-3 content in the fillet that benefits human health. Numerous vegetable oils (VOs) have been tested and adopted to reduce FO inclusion in aquafeeds, which can maintain or even enhance growth performance of farmed fish (Turchini, Trushenski & Glencross, 2019; Kwasek, Thorne-Lyman & Phillips, 2020; Cottrell et al., 2020). Among these VOs, rapeseed, soybean, palm and sunflower oils have been frequently used replace FO without negatively affecting fish growth (Turchini, Torstensen & Ng, 2009; Hixson, Parrish & Anderson, 2014b).

However, only a small proportion of FO can be replaced by VOs due to their lack in omega-3 (n-3) long-chain polyunsaturated fatty acids (LC-PUFA), particularly eicosapentaenoic (EPA; 20:5n-3) and docosahexaenoic (DHA; 22:6n-3) acids. On the other hand, VOs are rich in omega-6 (n-6) and omega-9 (n-9) PUFA, mainly linoleic acid (LA; 18:2n-6), and oleic acid (OA; 18:1n-9). Therefore, a consequence of high replacement levels of FO with VOs would be a reduction of EPA and DHA levels in cultured fish tissues that can eventually, compromise the animal’s health (Turchini, Torstensen & Ng, 2009) and the nutritional value of fish as food (Karakatsouli, 2012). Omega-3 LC-PUFA typically enter the human diet through the consumption of oily fish (Ruiz-Lopez et al., 2014b). Therefore, the challenge for the aquaculture industry is not to simply replace FO with alternative lipid sources, such as VOs, but to find sources with a the fatty acid profile that mimics that of pelagic oceanic fish that is typically consumed by farmed fish.

The oilseed plant, *Camelina sativa*, is a member of the Cruciferae (Brassicaceae) family and has potential to replace a higher proportion of FO in aquafeeds, which gives it a competitive advantage over other commercial oilseed crops, e.g., rapeseed and sunflower oils (Budin, Breene & Putnam, 1995). In particular, *C. sativa* seeds can produce oil containing up to 40% of the omega-3 precursors *α*-linolenic acid (ALA; 18:3n-3) and LA (Budin, Breene & Putnam, 1995; Eidhin, Burke & O’Beirne, 2003). The Camelina oil (CO) also contains high levels of PUFA and MUFA (monounsaturated fatty acids) and high amounts of antioxidants, e.g., γ-tocopherol. Lastly, CO represents a sustainable alternative to FO in aquafeeds due to its low environmental footprint (Budin, Breene & Putnam, 1995).

The use of CO from both wild type and transgenic *C. sativa* plants has been widely investigated in the last decade in diets for different fish species and at different replacement levels of FO (Hixson, Parrish & Anderson, 2013; Hixson, Parrish & Anderson, 2014a; Hixson, Parrish & Anderson, 2014b; Hixson & Parrish, 2014; Haslam et al., 2015; Betancor et al., 2015b; Betancor et al., 2015a; Betancor et al., 2016a; Betancor et al., 2016b; Betancor et al., 2018; Toyes-Vargas et al., 2020). Genetically modified *C. sativa* is capable of producing EPA or even both EPA and DHA in its seeds due to the insertion of five microalgal genes codifying for fatty acyl desaturase and elongase that are involved in n-3 LC-PUFA biosynthesis (Ruiz-Lopez et al., 2014a).

Given the scarce desaturation and elongation capacity of most marine fish species to produce EPA and DHA starting from ALA, efforts have focused on enhancing the composition of CO to include these important fatty acids. In comparison to marine fish species, salmonids usually show greater endogenous capacity to synthesize EPA and DHA from ALA (Hixson, Parrish & Anderson, 2014b). Historically, marine fish have inhabited environments and consumed pelagic fish rich in n-3 LC-PUFA, therefore, they have had no evolutionary pressure to retain the ability to endogenously produce EPA and DHA. In contrast, phytoplankton in freshwater are characterized by having high levels of LA and ALA, moderate levels of EPA and low levels of DHA, which has contributed to maintaining high selective pressures on freshwater fish to produce DHA (Tocher, 2010). High levels of n-3 LC-PUFA in CO are vital for effective replacement of FO in diets for marine species, such as sea bream, that cannot endogenously produce DHA and EPA and rely on their inclusion in the diet.

Alternative ingredients must also be tested for the modulation of fish intestinal microbiota and ramifications on fish health. Gut microbiota plays an important role in key functions in fish, including the supply of nutrients, intestinal integrity and interactions with the immune system (Llewellyn et al., 2014; Ringøet al., 2016). As suggested by Castro et al. (2019), the different fatty acid composition of VO, with respect to FO, could modify the lipid composition, function and fluidity of intestinal cell membranes. Consequently, this could affect bacteria adherence to enterocytes, alter metabolic processes and modify the intestinal microbiota profile.

Due to advances from DNA sequencing technologies in the last two decades, researchers can utilize next-generation sequencing (NGS) to investigate the microbes in and on the outside of fish for both basic and applied applications. Recently, several studies focused on gut microbiome have been conducted in a wide range of fish species (Rimoldi et al., 2019; Rimoldi et al., 2020b; Terova et al., 2019; Legrand et al., 2020; Blaufuss et al., 2020; Pérez-Pascual et al., 2020; Zhao et al., 2020; Panteli et al., 2020). However, despite the large number of studies, the effects of replacement of FO with novel lipid sources on fish gut microbiota structure have not been sufficiently examined (Piazzon et al., 2017; Torrecillas et al., 2017; Castro et al., 2019; Magalhães et al., 2020). Furthermore, few studies have used NGS to characterize the gut microbiota of gilthead sea bream (*Sparus aurata*) in response to diet (Estruch et al., 2015; Parma et al., 2016; Parma et al., 2020; Piazzon et al., 2017; Rimoldi et al., 2018; Rimoldi et al., 2020a; Piazzon et al., 2020), and even less have described the functional profile of gut microbiome in this species (Piazzon et al., 2019; Piazzon et al., 2020). Indeed, dietary changes can affect the structure of fish intestine’s associated microbial community (what’s there?) but, to understand the intrinsic processes that lead to similar functionality, it is necessary to search the connections between individual microbiota (what are they doing?) and the corresponding metabolic phenotype.

The aim of the present study was to determine the effects of replacing FO with CO on the growth performance, fillet fatty acid composition and gut microbiota of gilthead sea bream. We used advanced nutritional techniques to characterize fatty acid profiles, the NGS Illumina platform to characterize microbial populations in the gut and the meta-genomic tool PICRUSt to determine the functional potential of the gut microbiome.

## Materials & Methods

### Ethics Statement

Animal handling during the feeding trial complied with the guidelines of the European Union Council (86/609/EU) and Spanish legislation (RD 53/2013). Bioethical Committee of the University of Las Palmas de Gran Canaria (OEBA-ULPGC 21/2018R1) approved protocols used in the present trial.

### Animals

A feeding trial was conducted using 600 gilthead sea bream (*Sparus aurata*) (32.92 ± 0.31 g mean initial weight; 12.15 ± 0.37 cm mean initial length), at the Parque Científico-Tecnológico Marino (PCTM) of the University of Las Palmas de Gran Canaria (Telde, Canary Island, Spain). Fish were purchased from a commercial fish hatchery (Spain). Fish were acclimatized for 4 weeks to dissolved oxygen levels between 6.6–6.1 ppm and water temperatures between 18.2–20.2 °C. Gilthead sea bream were randomly allocated to twelve fiberglass, circular tanks of 250 L. Three tanks per diet were used with 50 fish each in a marine flow-through system with natural photoperiod (12L/12D). Fish were fed to apparent satiation 3 times per day, 6 days per week for 90 days.

### Diets and feeding trial

Four isonitrogenous, isolipidic, and isocaloric diets were formulated: Diet 1, control diet (100% FO); Diet 2 (20% CO, 80% FO); Diet 3 (40% CO, 60% FO); and Diet 4 (60% CO, 40% FO) (Table 1). The CO used in the trial was not derived from genetically modified *Camelina sativa* since the use of transgenic plants in aquafeed is not allowed in Italy. Naturalleva, VRM S.r.l (Cologna Veneta, Italy), manufactured the diets. Non-defatted fishmeal was employed as the major protein source to ensure essential fatty acid requirements of gilthead sea bream. The mash of each diet was extruded to a 3.0 mm pellet using a single-screw extruder (X-165,Wenger USA) before vacuum coating (La Meccanica vacuum coater, Italy). The vacuum coating process was divided into 4 phases: blend oil addition, air removal (200 mbar), mixing, and slow back to atmospheric pressure (1 min time).

**Table 1:** Main ingredients and proximate composition of the experimental feeds.

	**Diets**			
**Feed ingredients (%)**	** 1 (ctrl)**	**2**	**3**	**4**
Fish meal	19.4	19.4	19.4	19.4
Soybean meal	10.1	10.1	10.1	10.1
Guar germ meal	12.7	12.7	12.7	12.7
Wheat	14.5	14.5	14.5	14.5
Corn gluten	18.6	18.6	18.6	18.6
Soy protein concentrate	3.8	3.8	3.8	3.8
Fish oil	15.3	12.4	9.3	6.0
Camelina oil	0.0	2.9	6.0	9.3
Vitamins and minerals^a^^,^^b^	5.6	5.6	5.6	5.6
**Proximate composition** (g/100 g dry matter)				
Protein	43	43	43	43
Lipid	21	21	21	21
Ash	6.5	6.5	6.5	6.5
Fiber	1.4	1.4	1.4	1.4
NSP^*^	20.6	20.6	20.6	20.6
Moisture	7.5	7.5	7.5	7.5

**Notes.**

a Vitamin premix (IU or mg/kg diet): DL-α tocopherol acetate 60 IU; sodium menadione bisulphate 5 mg; retinyl acetate 15,000 IU; DL-cholecalciferol 3000 IU; thiamine 15 mg; riboflavin 30 mg; pyridoxine 15 mg; vitamin B12 0.05 mg; nicotinic acid 175 mg; folic acid 500 mg; inositol 1,000 mg; biotin 2.5 mg; calcium pantothenate 50 mg.

b Mineral premix (g or mg/kg of diet) bi-calcium phosphate 500 g, calcium carbonate 215 g, sodium salt 40 g, potassium chloride 90 g, magnesium chloride 124 g, magnesium carbonate 124 g, iron sulphate 20 g, zinc sulphate 4 g, copper sulphate 3 g, potassium iodide 4 mg, cobalt sulphate 20 mg, manganese sulphate 3 g, sodium fluoride 1 g.

* NSP, Non Starch Polysaccharide

### Lipid and fatty acid methyl esters (FAME) analysis of feeds and fish fillets

Approximately 0.07–0.1 g of finely grinded feed and 0.2 g of fillet were weighted and extracted to determine the total lipid content. All samples were analyzed in triplicate. A mixture of chloroform/methanol (2:1 v/v) with 0.01% BHT (butylated hydroxytoluene) was added to each sample, which was then homogenized using an Ultra-turrax (T25 Digital Ultra-turrax, IKA^®^, Germany). Lipid quantity was determined by a gravimetrical method followed by filtration using anhydrous sodium sulphate and evaporation to dryness under a nitrogen atmosphere (Folch, Lees & Sloane Stanley, 1957). The methylation of fatty acids was carried out following Christie’s method (William & Xianlin). A methanol solution containing 1% sulphuric acid was used followed by an incubation of the sample at 50 °C for 16 h. Then, the methylated fatty acids were separated by gas chromatography (GC-14A; Shimadzu) (Izquierdo et al., 1992) equipped with a Supelcowax 10 capillary GC column (30 m × 0.32 mm ID, 0.25 µm film thickness), quantified by flame ionizator detector (FID) and identified by comparison to external standards (EPA 28, Nippai, Ltd. Tokyo, Japan). Helium was used as a carrier gas and the injection volume was set at 1 µl. The following thermal conditions were used to discriminate each fatty acid: an initial temperature of 170 °C for 2 min and then increased to 220 °C at a rate of 2 °C min^−1^ for 15 min. The temperature of the injector and the flame ionization detector was 250 °C.

### Fish intestine and feed sampling for microbiota analyses

At the end of the feeding trial, nine fish per diet were euthanized with an overdose of clove oil, dissected and intestinal content/faeces (36 samples) and mucosa (36 samples) were collected in order to analyse the luminal and mucosa-associated microbiota, respectively, as described by Terova et al. (2019) and Rimoldi et al. (2019). Both faeces and mucosa samples were stored in Xpedition Lysis/Stabilization Solution (Zymo Research) at room temperature up to 48 h until bacterial DNA extraction. Additionally, four aliquots of 200 mg from each extruded diets were collected and stored at 1 °C for the microbiota analysis.

### Bacterial DNA extraction

Bacterial DNA extraction was carried out at the University of Insubria, Varese, Italy, following the protocol described in details by Rimoldi et al. (2020b). Specifically, the bacterial DNA was extracted from 4 samples of each feed, 9 samples of intestinal content/feaces and 9 samples of intestinal mucosa per dietary group (88 samples in total) using the DNeasy PowerSoil Kit (Qiagen, Italy) and homogenized using a TissueLyser II (Qiagen, Italy) set at 25 Hz for 2 min. The extracted DNA was spectrophotometrically quantified and stored at minus 20 °C until the molecular analysis were performed.

### 16S Illumina library construction and High-throughput sequencing

The metagenomics analysis was carried out at the University of Stirling. The v4 region of the 16S rRNA was amplified by PCR. The reaction mix consisted of 2 µl template (5 ng of DNA), 1.25 µl (10 µM) of each forward primer (515F; GTGYCAGCMGCCGCGGTAA) and reverse primer (806R; GGACTACNVGGGTWTCTAAT) (Caporaso et al., 2011) with Illumina adapter,12.5 µL of 2x NEBNext Ultra II Q5 Master Mix (New England Biolabs Ltd., Hitchin, UK), and nuclease free water to final volume of 25 µl. The PCR conditions were as follows: 98 °C for 60 s followed by 30 cycles of 98 °C for 10 s, 53 °C for 10 s and 65 °C for 45 s with a final step of 65 °C for 5 min. A 1% agarose gel alongside negative controls (nuclease free water) confirmed the presence of each amplicon band. Axygen AxyPrep Mag PCR clean up kit (Corning Inc., Corning, NY, USA) was used to purify samples using magnetic beads (0.8:1 ratio) and two washes of 70% EtOH, according to the manufacturer’s instructions. Tris 10 mM (Qiagen Ltd.) was used to elute the sample and a second PCR was performed with the above conditions, except only for 10 cycles and the primers were tagged with 8 bp unique indices from the Nextera XT DNA Library Preparation kit (Illumina Inc., Cambridge, UK). Axygen magnetic beads (Corning Inc.) and a Quibit 2.0 fluorimeter (Thermo Fisher Scientific) were used to purify and quantify the samples, which were then diluted to 10 nM with Tris buffer and pooled at equimolar concentrations. The amplicon library was sequenced on the Illumina MiSeq platform at the University of Stirling (Stirling, UK) with a MiSeq Reagent kit v2 of 500 cycles (Illumina Inc.).

### 16S amplicon sequencing data analysis

Mothur version 1.42.3 (Schloss et al., 2009) was used to analyse the 16S rRNA sequences according to the MiSeq SOP (https://www.mothur.org/wiki/MiSeq_SOP) (Kozich et al., 2013). Filtered sequences were between 250–300 bp, <8 consecutive bp and were within the v4 region. The SILVA reference database version 123 (Quast et al., 2013) and VSEARCH (Rognes et al., 2016) were used to align the sequences and remove chimeras. The RDP Bayesian Classifier trainset version 16.0 at a cut-off of 80% (Cole et al., 2014) was used and taxon resembling chloroplasts, mitochondria, unknowns, archaea and eukaryotes were removed. In addition, *Undibacterium* (genus level) was removed since it comprised 95% of OTUs in the blank samples and it is a known laboratory contaminant (Salter et al., 2014). Sequences were clustered to the genus level (0.03) and subsampled (normalised) to 10,171 sequences per samples. Seven mucosa samples were not included (two M0, three M20, one M40 and one M60) since mitochondria were >50% of sequences, which meant there were too few sequences per sample. The raw 16S rRNA sequence reads were deposited in the Sequence Read Archive of NCBI and made publicly available under BioProject Accession number PRJNA657669.

### 16S PICRUSt metagenome analysis

Phylogenetic Investigation of Communities by Reconstruction of Unobserved States (PICRUSt) was used to generate predictive pathways based on Greengenes classified OTU table (version 13.8.99) (DeSantis et al., 2006) and Kyoto Encyclopaedia of Genes and Genomes (KEGG) database (Kanehisa & Goto, 2000) according to the PICRUSt tutorial (https://github.com/LangilleLab/microbiome_helper/wiki/PICRUSt-tutorial) (Langille et al., 2013). The OTU copy number was normalised across samples and extended error plots were made using Statistical Analysis of Taxonomic and Functional Profiles (STAMP) with Welch’s two-sided *T*-test with 95% confidence intervals (Parks et al., 2014).

### Statistics

Data were reported by means and standard deviation. The lipid profile was measured on a sample size of nine fish per diet (*n* = 9). Percentage data were subjected to arcsin square root transformation before statistical analysis. Data were tested for normality and homogeneity of variances Shapiro–Wilk’s and Levene’s test, respectively. Differences between groups were analysed by one-way ANOVA followed by a Tukey’s post hoc test. When the data were not normally distributed, the Kruskal-Wallis’s test was performed followed by Dunn’s post hoc test. All analyses were performed with Past3 software (Hammer et al., 2001).

With regard to microbiota analysis, normal distribution and homogeneity of each dataset were determined using Shapiro–Wilk and Levene tests in Rstudio (RCT, 2019). If needed, data were normalized by log, square-root or arcsine transformation. One-way Analysis of Variance (ANOVA) was used to determine the effect of diet, sample type and gut section on growth performance, fatty acid and alpha diversity of gut bacteria. *P*-values between treatments were determined using Least Square Means (lsmeans) with Tukey adjustment for multiple comparisons. Alpha diversity was based on Good’s coverage, observed species, Shannon diversity (non-parametric) and Chao-1 richness indices. Beta-diversity was analysed using a Bray–Curtis distance matrix (sqrt-transformed) and significance of treatments were determined using Analysis of Similarity (ANOSIM) with the adonis function (vegan package) in R (Oksanen et al., 2014) and plotted using 2D non-metric multidimensional scaling (NMDS). Linear discriminant analysis Effect Size (LefSe) was used to identify OTUs that explain differences between treatments using Kruskal-Wallis tests and a Linear Discriminant Analysis (LDA) threshold of 3.0 (Segata et al., 2011). *P*-values below 0.05 were considered significant.

## Results

### Fish growth performance

There were no significant differences in the mean initial weights between the feeding groups (Table 2). After 90 days of feeding, fish fed with diets 2 and 3 in which CO, replaced 20% and 40% of FO, respectively, showed the same growth as the control fish fed with 100% FO. The growth of fish fed with diet 4 in which CO replaced 60% of FO was similar to fish fed diets 1 and 3, but significantly lower (*p* < 0.05) in comparison fish fed diet 2. No significant differences between groups were detected for FCR (Feed Conversion Rate) and SGR (Specific Growth Rate).

All the experimental feeds were well accepted (no palatability issues) by gilthead sea bream that grew well, almost tripling their initial weight at the end of the feeding trial. Moreover, no mortality was observed during the 90 days of experiment (final survival = 100% in all dietary treatments).

### Lipid content and FAME profile of fillets

The lipid profile of gilthead sea bream fillets were similar between the four dietary groups in regards to total saturated (SFA), monounsaturated (MUFA), PUFA (n-3 and n-6) and the ratio of *n* − 3∕*n* − 6 (Table 3). For a few individual fatty acids, levels differed between the dietary groups, especially in fish fed diet 4 (60% CO). In particular, the highest level of EPA (20:5n-3) was recorded in fish fed with 0% CO (diet 1), whereas fish fed with 60% CO (diet 4) showed the lowest level (Table 3). The highest level of 18:3n-3 (α-linolenic acid) was found in fish fed the 60% CO diet and corresponded to a high level in the CO diet (Table 4). Moreover, the 60% CO-fed fish showed the highest level of linoleic acid (18:2n-6) in contrast to the level detected in the 0% CO-fed fish (13.94 ± 1.27 vs 9.39 ± 1.19; *p* < 0.01). Similarly, the 40% CO-fed fish (diet 3) showed significantly higher level of 18:2n-6 than the 0% CO-fed fish (11.40 ± 3.63 vs 9.39 ± 1.19; *p* < 0.05) (Table 3).

**Table 2:** Effect of Camelina oil on gilthead sea bream growth performance. Values are presented as means (±std. dev). Superscript letters indicate statistical significance between dietary groups. The significance was determined using a One-Way ANOVA and a Tukey Test with a *p* value < 0.05.

**Diet**	**Initial weight**	**Final weight**	**SGR (% ⋅ day**^−1^**)**	**FCR**
1	32.80 ± 1.97	87.88 ± 12.93^ab^	0.98 ± 0.07	1.25 ± 0.08
2	32.81 ± 2.01	89.27 ± 14.89^a^	1.00 ± 0.04	1.24 ± 0.08
3	33.02 ± 2.15	85.02 ± 11.94^ab^	0.95 ± 0.02	1.33 ± 0.06
4	33.19 ± 2.06	83.86 ± 11.65^b^	0.94 ± 0.03	1.36 ± 0.12

**Table 3:** Lipid content and fatty acid profile (percentage of total fatty acids) of fish fillets.

	**Fish dietary groups**				*ANOVA *p*-value*	*Kruskal–Wallis *p*-value*
	**1** (0%CO)	**2** (20%CO)	**3** (40%CO)	**4** (60%CO)		
Lipid content (% wet weight)	5.64 ± 1.74	5.70 ± 1.37	4.72 ± 1.80	4.18 ± 0.98	0.09	
C14:0	2.65 ± 1.09	2.86 ± 1.52	1.81 ± 0.78	1.88 ± 0.43		0.12
C15:0	0.38 ± 0.26^B^	0.39 ± 0.14^A^	0.29 ± 0.09^B^	0.35 ± 0.19^B^		<0.001
C16:0	14.66 ± 4.00	13.86 ± 6.43	11.86 ± 5.01	11.92 ± 2.84	0.65	
C17:0	0.51 ± 0.13	0.60 ± 0.29	0.55 ± 0.31	0.41 ± 0.15		0.25
C18:0	4.31 ± 0.91	4.06 ± 1.12	3.42 ± 1.43	4.33 ± 0.47		0.63
C20:0	0.45 ± 0.18	0.86 ± 1.34	0.36 ± 0.26	0.78 ± 1.14		0.50
Total saturated	22.97 ± 5.54	22.63 ± 5.15	18.29 ± 4.48	19.67 ± 4.49	0.99	
C14:1*n*−7	0.17 ± 0.14^AB^	0.24 ± 0.18^A^	0.05 ± 0.08^B^	0.14 ± 0.17^AB^	0.006	
C14:1*n*−5	0.31 ± 0.18	0.44 ± 0.40	0.32 ± 0.33	0.29 ± 0.26		0.82
C15:1*n*−5	0.14 ± 0.14	0.43 ± 0.41	0.39 ± 0.53	0.18 ± 0.22		0.06
C16:1*n*−7	4.98 ± 1.47	4.84 ± 1.96	3.50 ± 1.35	3.30 ± 0.85	0.06	
C18:1*n*−5	0.33 ± 0.24	0.41 ± 0.32	0.50 ± 0.52	0.32 ± 0.26	0.78	
C18:1*n*−7	3.19 ± 0.34^A^	2.81 ± 0.61^AC^	2.46 ± 0.38^BC^	2.32 ± 0.19^BC^		<0.001
C18:1*n*−9	22.89 ± 2.30^Aab^	19.58 ± 6.72^aB^	18.41 ± 5.81^ABb^	20.89 ± 2.40^Aab^		<0.001
C20:1*n*−9	0.40 ± 0.17	0.33 ± 0.20	0.53 ± 0.54	0.37 ± 0.23		0.57
C20:1*n*−7	1.64 ± 0.38	1.31 ± 0.63	1.38 ± 0.34	1.55 ± 0.36		0.39
C22:1*n*−11	1.43 ± 0.67	1.54 ± 1.03	1.24 ± 0.80	0.97 ± 0.32		0.22
C22:1*n*−9	0.66 ± 0.31	0.62 ± 0.19	0.59 ± 0.42	0.67 ± 0.28	0.87	
Total monoenes	36.14 ± 6.68	32.55 ± 5.69	29.37 ± 5.32	31.00 ± 6.08		0.97
C18:2*n*−6	9.39 ± 1.19^Bb^	9.21 ± 2.98^Bab^	11.40 ± 3.63^ABa^	13.94 ± 1.27^Aab^	<0.001	
C18:3*n*−6	0.00 ± 0.00^Bab^	0.00 ± 0.00^Bab^	0.29 ± 0.25^ABb^	0.70 ± 0.39^Aa^		<0.001
C20:2*n*−6	0.44 ± 0.22	0.83 ± 1.07	0.43 ± 0.30	0.51 ± 0.20	0.38	
C20:3*n*−6	0.50 ± 0.26	0.81 ± 1.23	0.38 ± 0.30	0.42 ± 0.30		0.53
C20:4*n*−6	0.95 ± 0.21	0.63 ± 0.31	0.78 ± 0.18	0.69 ± 0.14		0.05
C22:5*n*−6	0.73 ± 0.46	1.35 ± 1.66	0.39 ± 0.28	0.51 ± 0.47		0.13
Total *n*-6 PUFA	12.01 ± 3.63	12.83 ± 3.49	13.67 ± 4.47	16.77 ± 5.46		0.63
C16:3*n*−3	0.21 ± 0.17	0.41 ± 0.32	0.49 ± 0.54	0.26 ± 0.22	0.37	
C16:4*n*−3	0.51 ± 0.12	0.60 ± 0.35	0.59 ± 0.47	0.50 ± 0.31		0.61
C18:3*n*−3	1.73 ± 0.25^B^	4.33 ± 1.14^BC^	8.57 ± 2.86^AC^	12.16 ± 1.79^AC^		<0.001
C18:4*n*−3	1.04 ± 0.25	1.06 ± 0.39	1.06 ± 0.22	1.10 ± 0.29	0.99	
C20:3*n*−3	0.25 ± 0.16^b^	0.39 ± 0.19^ab^	0.48 ± 0.17^ab^	0.60 ± 0.33^a^	0.01	
C20:4*n*−3	1.01 ± 0.73	0.78 ± 0.20	0.73 ± 0.23	0.73 ± 0.21		0.55
C20:5*n*−3	6.86 ± 1.79^a^	5.57 ± 1.68^a^	5.34 ± 1.11^ab^	4.25 ± 0.97^b^		0.006
C22:5*n*−3	2.61 ± 1.06	2.99 ± 1.81	2.44 ± 0.72	1.92 ± 0.42	0.31	
C22:6*n*−3	8.96 ± 3.62	7.09 ± 3.51	7.44 ± 1.46	6.40 ± 1.75	0.33	
Total *n*-3 PUFA	23.18 ± 3.16	23.22 ± 2.54	27.14 ± 3.24	27.92 ± 3.92		0.98
Total PUFA	35.19 ± 3.24	36.05 ± 2.84	40.81 ± 3.64	44.69 ± 4.44		0.98
EPA + DHA	15.82 ± 5.27	12.66 ± 5.15	13.09 ± 2.01	10.91 ± 2.66	0.10	
*n* − 3∕*n* − 6 ratio	1.93 ± 0.48	1.81 ± 0.48	1.99 ± 0.55	1.66 ± 0.32	0.35	

**Notes.**

Data expressed as means ± SD (*n* = 9).

Different capital and small letters within row show significant differences among diets at *p* < 0.01 and *p* < 0.05, respectively.

Statistical differences were determined by one-way ANOVA with Tukey’s comparison test and by Kruskal–Wallis with Dunn’s test.

**Table 4:** Fatty acid compositions of the oils and experimental diets (% of total fatty acid detected).

					**Diets**	
**Fatty acid**	**FO**	**CO**	**0%CO**	**20%CO**	**40%CO**	**60%CO**
C14:0	6,82	0,04	6,20	5,28	4,00	2,84
C15:0	0,48	0,00	0,42	0,37	0,28	0,21
C16:0	16,55	5,94	19,40	18,00	15,40	12,90
C17:0	0,06	0,01	0,26	0,24	0,20	0,16
C18:0	3,47	2,69	3,60	3,95	4,07	4,21
C20:0	0,41	0,87	0,38	0,38	0,37	0,37
Total saturated	27,79	9,55	30,26	28,22	24,32	20,69
C14:1*n*−5	0,03	0,00	trace	trace	trace	trace
C15:1*n*−5	0,00	0,00	0,03	0,00	0,01	0,02
C16:1*n*−5	1,11	0,13	7,70	6,70	5,12	3,60
C18:1*n*−9	15,36	16,46	19,00	16,80	17,30	18,40
C18:1*n*−7	0,92	0,20	2,62	2,54	2,52	2,02
C18:1*n*−5	ND	ND	0,17	0,23	0,12	0,08
C20:1*n*−7	ND	ND	1,97	0,91	1,72	1,47
C20:1*n*−5	ND	ND	0,28	0,20	0,18	0,13
C20:1*n*−9	3,43	9,05	2,00	1,97	1,80	1,70
C22:1*n*−11	2,47	0,77	1,76	1,61	1,23	0,97
C22:1*n*−9	0,59	1,38	0,60	1,41	0,32	0,29
Total monoenes	23,91	27,99	36,13	32,37	30,32	28,68
C18:2*n*−6	3,78	21,32	7,90	9,70	12,90	16,00
C18:3*n*−6	0,26	0.02	0,52	0,45	0,34	0,27
C20:2*n*−6	0,36	1,45	0,35	0,25	0,19	0,15
C20:3*n*−6	0,16	0,63	0,10	0,09	0,07	trace
C20:4*n*−6	0,83	0,00	0,80	0,71	0,54	0,38
Total *n*-6 PUFA	23,21	6,24	9,67	11,20	14,04	16,80
C16:3*n*−3	ND	ND	0,14	0,24	0,11	0,06
C16:4*n*−3	ND	ND	0,54	1,28	0,67	0,74
C18:3*n*−3	1,68	37,01	1,99	8,90	17,20	24,20
C18:4*n*−3	2,22	0,01	2,17	1,87	1,43	0,98
C20:3*n*−3	0,03	0,03	0,12	0,13	0,13	0,13
C20:4*n*−3	0,52	0,00	0,53	0,51	0,30	0,30
C20:5*n*−3	12,92	0,00	13,00	11,40	8,60	5,80
C22:5*n*−3	1,65	0,00	1,34	1,24	0,93	0,64
C22:6*n*−3	9,16	0,00	6,70	5,90	4,50	3,12
Total *n*-3 PUFA	27,87	37,72	26,53	31,47	33,87	35,97
Total PUFA	51,08	43,96	36,20	42,67	47,91	52,77
EPA + DHA	22,08	0,00	19,70	17,30	13,10	8,92
*n*3/*n*6 ratio	5.53	1.70	2,74	2,81	2,41	2,14

**Notes.**

Diets: 0% CO, control diet based on fish oil and without camelina oil (CO); 20% CO, diet contained 20% of camelina oil; 40% CO, diet contained 40% of camelina oil; 60% CO, diet contained 60% of camelina oil.

The content of three MUFA, namely C14:1n-7; C18:1n-7; and C18:1n-9 were significantly different between diet groups. The 20% CO-fed fish showed the highest level of C14:1n-7 compared to 40% CO-fed fish (0.24 ± 0.18 vs 0.05 ± 0.08; *p* < 0.01), but no differences were found in 0% and 60% CO-fed fish. With regard to C18:1n-7, fish fed 0% CO had higher content of this fatty acid in the fillet compared to 40 and 60% CO-fed fish (3.19 ± 0.34 vs 2.46 ± 0.38 and 2.32 ± 0.19; *p* < 0.01), but similar levels compared to 20% CO-fed fish (3.19 ± 0.34 vs 2.81 ± 0.61). The 20% and 60%-fed fish had higher levels of C18:1n-9 compared to 20% CO-fed fish (22.89  ± 2.30 and 20.89 ± 2.40 vs 19.58 ± 6.72; *p* < 0.01), but similar levels as 40% CO-fed fish.

### Alpha-diversity of bacteria in the gut and diet

**Figure 1:**
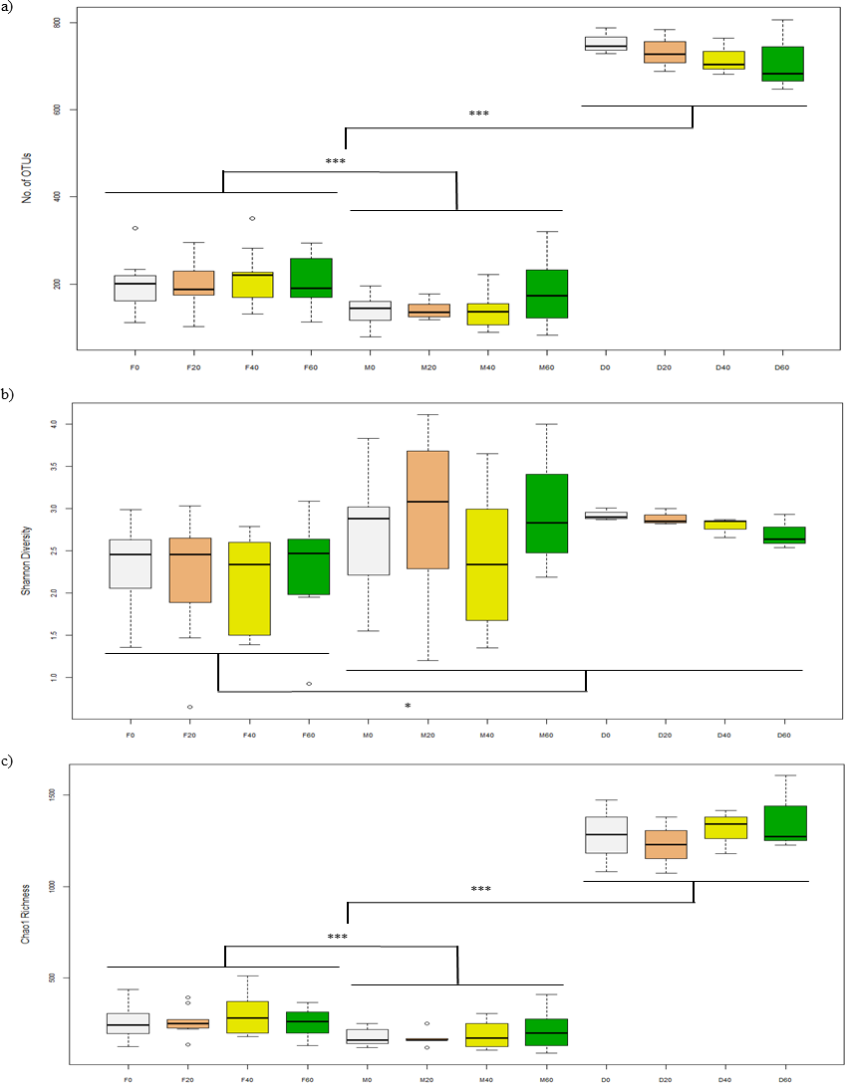
Alpha-diversity of OTUs based on (A) No. of OTUs, (B) Shannon, and (C) Chao1 indices from the faeces/digesta (F), mucosa (M) and diets (D) of gilthead sea bream where fish oil was replaced with 0, 20, 40 and 60% camelina oil.

After filtering sequences, the Illumina MiSeq generated 2.7 million sequences for a library of 82 samples (32,721 reads per sample) and was further subsampled to 10,171 sequences per sample. Alpha-diversity of bacteria in the faeces (F0, F20, F40, F60) was not significantly different between diets in terms of number of OTUs (*p* = 0.940), Shannon (*p* = 0.926) and Chao1 (*p* = 0.780) indices (Figs. 1A–1C). For mucosa (M0, M20, M40, M60), there were no differences between diets for the above indices (*p* = 0.535, 0.521 and 0.930) and the same was found for diet (D0, D20, D40, D60) (*p* = 0.760, 0.234 and 0.791). Between each sample type, there were significant differences in terms of number of OTUs (*p* = 0.030) (Fig. 1A), Shannon (*p* = 0.003) (Fig. 1B) and Choa1 (*p* < 0.001) (Fig. 1C). All three types of sample were different for number of OTUs, Shannon and Chao1 (*p* < 0.05), except Shannon diversity was similar between mucosa and diet (*p* = 0.874).

### Beta-diversity of bacteria in the gut and diet

**Figure 2:**
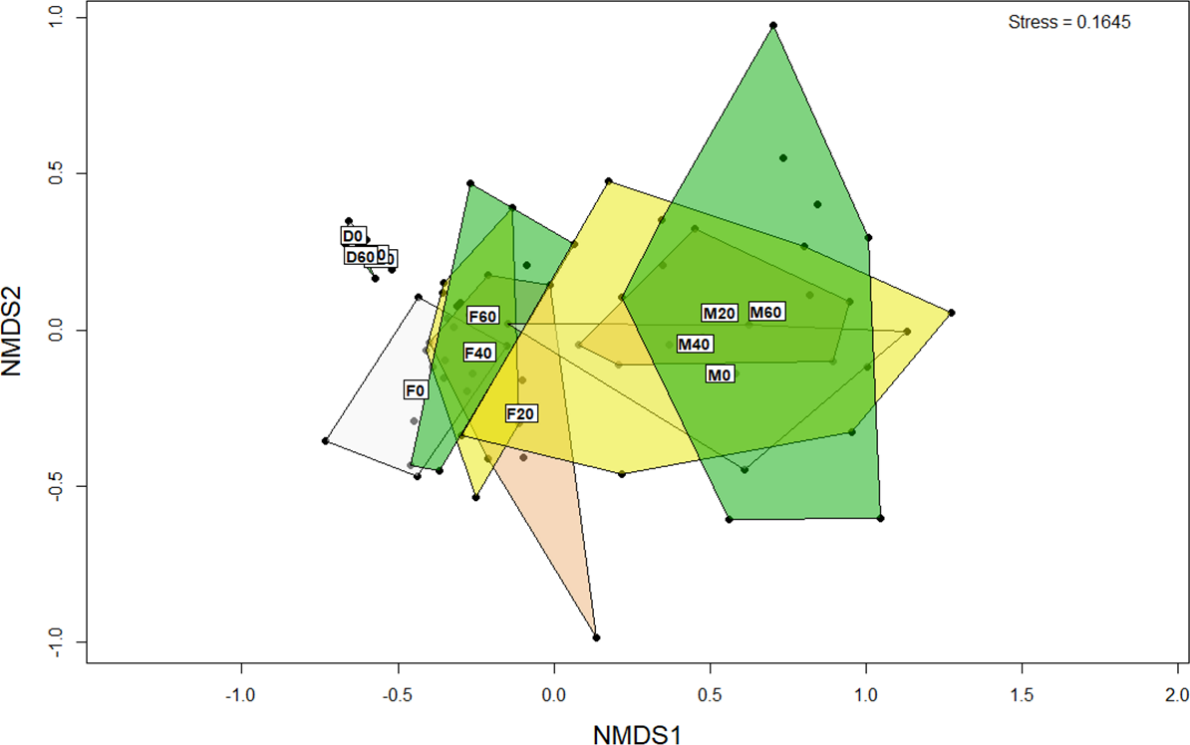
Non-metric multidimensional scaling (NMDS) plot of OTUs (genus level) from the faeces/digesta (F), mucosa (M) and diets (D) of gilthead sea bream where fish oil was replaced with 0, 20, 40 and 60% camelina oil.

**Figure 3:**
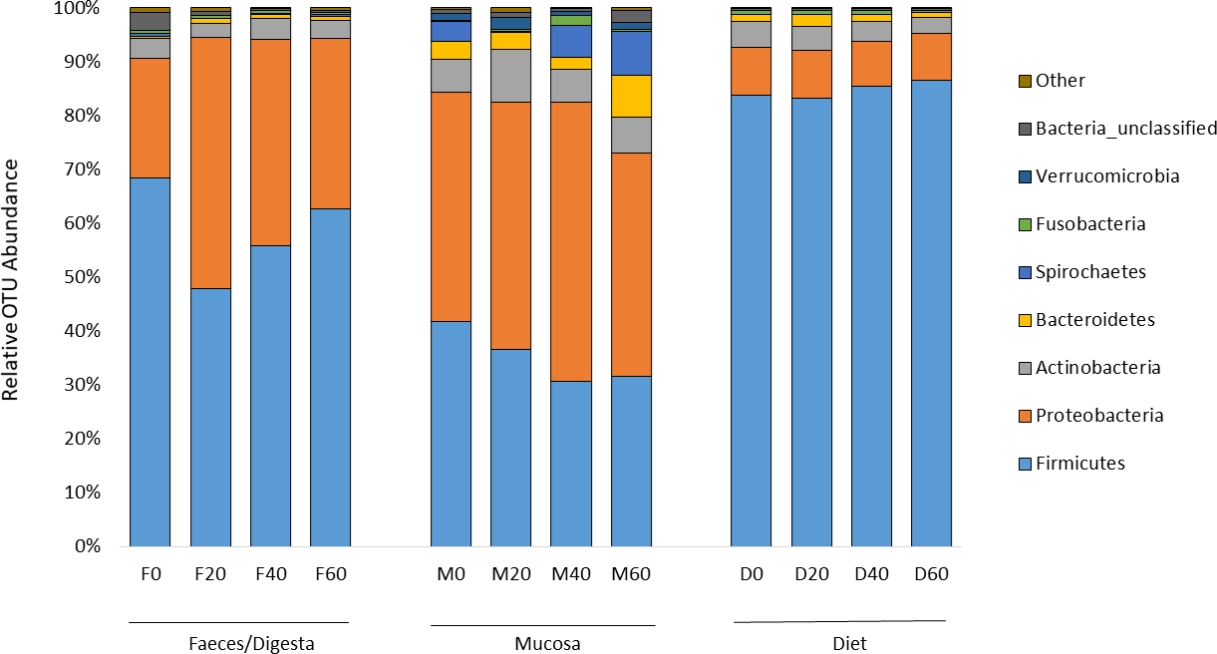
Percentage of OTUs (phyla level) from the faeces/digesta (F), mucosa (M) and diets (D) of gilthead sea bream where fish oil was replaced with 0, 20, 40 and 60% camelina oil.

**Figure 4:**
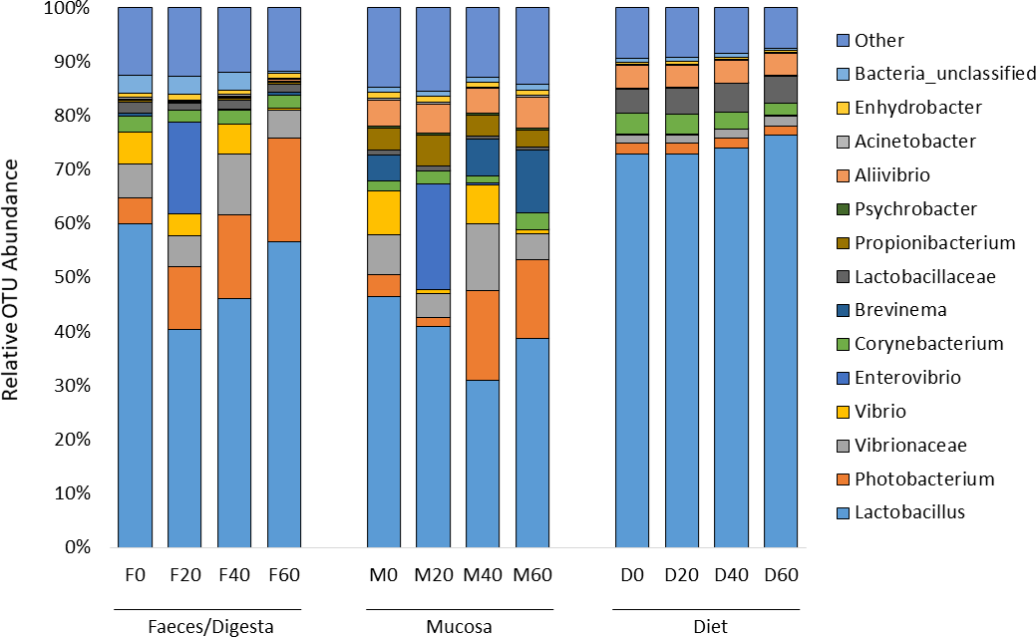
Percentage of OTUs (genus level) from the faeces/digesta (F), mucosa (M) and diets (D) of gilthead sea bream where fish oil was replaced with 0, 20, 40 and 60% camelina oil.

ANOSIM analysis revealed that the composition of bacteria was significantly different between sample types (faeces vs mucosa, *p* < 0.001), but it was not modulated by diet neither in faeces nor in mucosa samples (*p* > 0.05). The non-metric multidimensional scaling (NMDS) confirmed the statistical analysis as shown in Fig. 2. At the phyla level, Firmicutes and Proteobacteria were the most dominant with a relative abundance up to 94% in faeces, 92% in mucosa and 95% in diet (Fig. 3). The ratio of Firmicutes:Proteobacteria was highest in the diets and lowest in the mucosa. Actinobacteria, Bacteroidetes, Spirochaetes and other bacteria made up the small remainder. At the genus level, *Lactobacillus* was the most dominant with up to 60% in faeces, 47% in mucosa and 77% in diets (Fig. 4). *Photobacterium*, *Vibrio*, *Enterovibrio*, *Corynebacterium*, *Brevinema* and other bacteria made up the remainder.

In regards to specific indicator OTUs, there were differences between dietary treatments based on LEfSe analysis. In faeces, genera of *Peptostreptococcus*, *Vagococcus* and *Massilia* increased in fish fed diets with 60% replacement with CO (Table 5). *Lactobacillus* was most associated with the 0% CO diet. In mucosa, there were more OTUs associated with diet type. Increased abundance of *Corynebacterium*, *Rubrobacter*, *Ruegeria*, *Acetobacter* and *Enhydrobacter* was found in fish fed diets with 60% replacement with CO (Table 6). Several genera in the phylum of Proteobacteria, such as *Psychrobacter*, were associated with the mucosa of fish fed the control diet (0% CO).

**Table 5:** Indicator OTUs identified by LEfSe. OTUs found in the faeces of S. aurata (*n* = 9) fed diets that replaced FO with 0% CO (Ctrl) and 60% CO.

**Phyla**	**Order**	**OTU**	**%CO Diet**	**LDA**	*P***-value**
Firmicutes	Bacillales	*Cerasibacillus*	0	3.27	0.003
	Clostridiales	*Peptostreptococcus*	**60**	3.40	<0.001
	Lactobacillales	*Lactobacillus*	0	4.87	0.010
		*Pediococcus*	0	3.78	0.007
		*Vagococcus*	**60**	3.16	0.001
Proteobacteria	Burkholderiales	*Massilia*	**60**	3.43	<0.001

**Table 6:** Indicator OTUs identified by LEfSe. OTUS found in the intestinal mucosa of gilthead sea bream (*n* = 9) fed diets that replaced fish oil with camelina oil (CO).

**Phyla**	**Order**	**OTU**	**%CO Diet**	**LDA**	*P***-value**
Actinobacteria	Corynebacteriales	*Corynebacterium*	**60**	3.82	<0.001
	Rubrobacterales	*Rubrobacter*	**60**	3.19	0.027
Firmicutes	Bacillales	*Exiguobacterium*	0	3.32	0.011
Proteobacteria	Alteromonadales	*Idiomarina*	0	3.84	0.001
	Cellvibrionales	*Haliea*	0	3.05	0.005
	Oceanospirillales	*Halomonas*	0	3.98	<0.001
		*Kangiella*	0	3.04	0.021
	Pseudomonadales	*Psychrobacter*	0	4.26	<0.001
	Rhodobacterales	*Loktanella*	0	3.34	0.029
		*Ruegeria*	**60**	3.52	0.008
	Rhodospirillales	*Acetobacter*	**60**	3.83	<0.001
		*Enhydrobacter*	**60**	3.92	0.009
	Salinisphaerales	*Salinisphaera*	0	3.08	0.004

### Metagenome predictive pathways of gut bacteria

**Figure 5:**
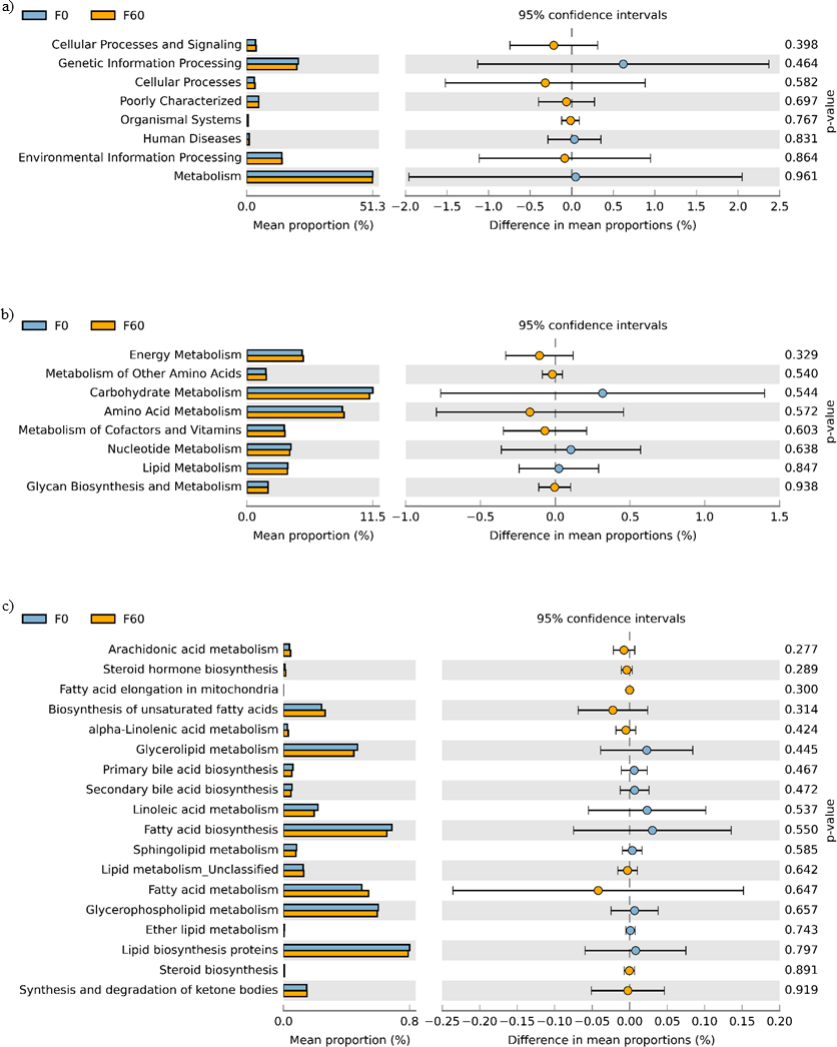
STAMP plots of predicted functional metagenomic pathways (PICRUSt) of OTUs in faeces/digesta (F) of gilthead sea bream where fish oil was replaced with 0 and 60% camelina oil under KEGG Levels 1 (A), 2 (B) and 3 (C).

**Figure 6:**
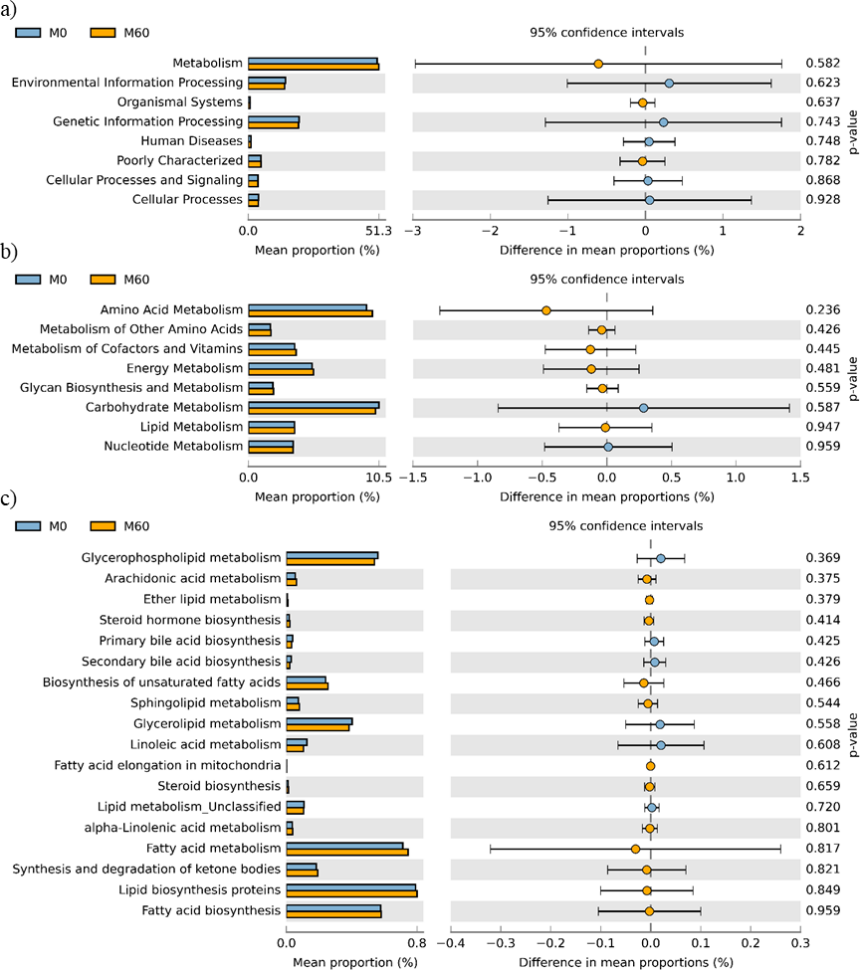
STAMP plots of predicted functional metagenomic pathways (PICRUSt) of OTUs in mucosa (M) of gilthead sea bream where fish oil was replaced with 0 and 60% camelina oil under KEGG Levels 1 (A), 2 (B) and 3 (C).

**Figure 7:**
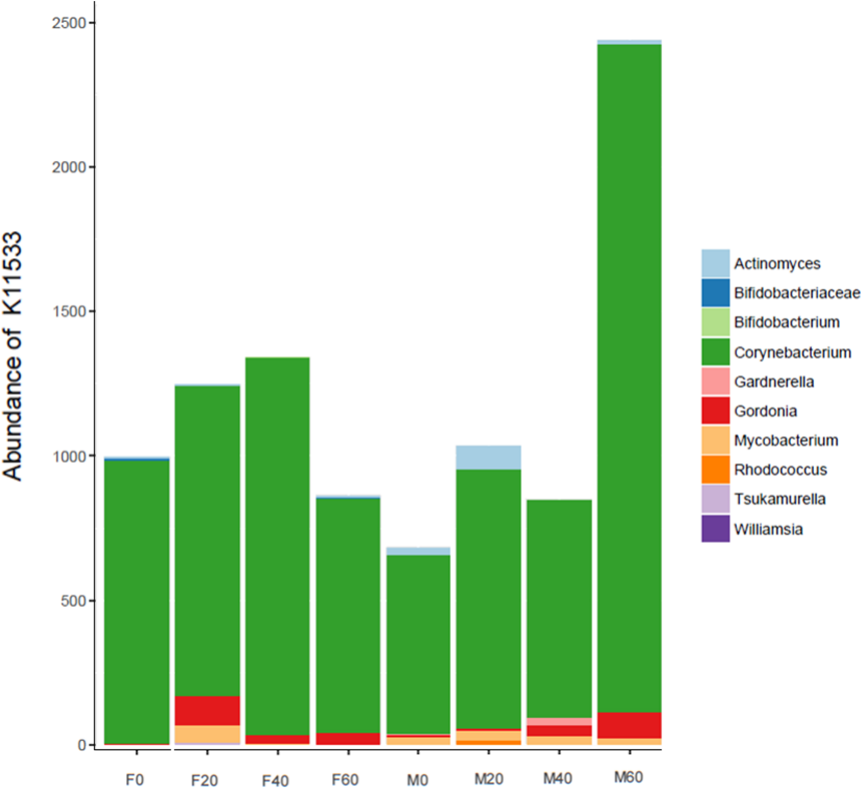
Contribution of bacterial OTUs to the abundance of K11533 (fatty acid synthase) in the faeces (F) and mucosa (M) of gilthead sea bream fed 0, 20, 40 and 60% CO.

No effect of replacing FO with 60% CO was found on the predictive pathways of bacteria from the faeces or mucosa (Figs. 5A–5C and 6A–6C). Metabolism accounted for 51% of the pathways, followed by genetic and environmental information processing. Metabolism of amino acids and carbohydrates contributed the most to the predictive pathways and to a lesser extent the metabolism of vitamins, energy and lipid. Within lipid metabolism, lipid biosynthesis proteins accounted for 0.8% of total pathways followed by fatty acid biosynthesis and metabolism of glycerophospholipid, fatty acids, glycerolipid and unsaturated fatty acids (Figs. 5C and 6C). Although not significant, metabolism of unsaturated fatty acids, arachidonic acid (20:4n-6) and alpha-linolenic acid (18:3n-3) were higher in fish fed 60% CO in both faecal and mucosal bacterial metagenome. Specifically, fish fed the 60% CO diet had an increased abundance of K11533 pathway (fatty acid synthase) attributed to *Corynebacterium* in the gut mucosa (Fig. 7). No significant differences were found in terms of metagenomic pathways between transient and resident intestinal communities. Of note, PICRUSt software is optimized for mammalian samples, consequently these results only reflect the metabolic potential of gut microbial communities in gilthead sea bream.

## Discussion

Reduced final weight of gilthead sea bream fed 60% CO (Table 2) demonstrated that this level of dietary inclusion is too high, although diets that replace 40% of FO with CO are feasible without negative effects on growth. This result contradicts a previous study on the same species where full replacement of FO by CO did not elicit differences in growth (Betancor et al., 2016a). In this previous study, the experimental diets were rich in fishmeal (FM; i.e., 49%), which may have compensated for the low levels of LC-PUFA in the diet. On the contrary, full replacement of FO with CO did not negatively affect growth of rainbow trout (*Oncorhynchus mykiss*) after 12-weeks (Hixson, Parrish & Anderson, 2014a). However, in another study by the same authors (Hixson, Parrish & Anderson, 2014b), the growth performance of Atlantic cod (family Gadidae), but not that of Atlantic salmon (*Salmo salar*) and rainbow trout (family Salmonidae), was negatively affected when fed diets that replaced 100% of FO with CO. As attested by Turchini, Torstensen & Ng (2009), a successful replacement of FO with terrestrial plant oil may be more readily achieved for fatty fish, such as salmonids, that have a higher and larger variety of fatty acids in their tissues than in lean fish, such as Atlantic cod (*Gadus morhua*), European sea bass (*Dicentrarchus labrax*), and gilthead sea bream.

In regards to transgenic CO, a study by Betancor et al. (2015b) fed post-smolt Atlantic salmon for 7-weeks with one of three experimental diets containing either FO, wild-type CO or transgenic CO as sole lipid source and showed contrasting results compared to our study. Atlantic salmon fed both wild and transgenic CO diets had similar final weight compared to salmon fed the FO diet. In a lean species such as European sea bass, the use of a high EPA+DHA genetically modified-*Camelina sativa* oil has instead been shown to promote adequate growth even at moderate dietary inclusion of FM (i.e., 25%) (Betancor et al., 2021). In the present study, FM was included at 19.4%, with no differences observed in growth with a 40% substitution of FO by CO. Other studies in the same species, did not find an effect on growth or feed utilization when FO was replaced up to 75% with VO at FM levels between 15–20% (Sánchez-Moya et al., 2020). The poorest growth in fish fed with 60% CO could be due to the presence of glucosinolates, which are responsible for the bitter or sharp taste of many cruciferous vegetables (Clarke, 2010) and are known to be prevalent in CO (Berhow et al., 2013). Indeed, poor palatability has been observed when using 100% CO substitution in European sea bass during the first two months of the experimental trial (Betancor et al., 2021).

We hypothesized that dietary CO alters the intestinal microbiota and its metabolic pathways. The NGS of gut microbiota in gilthead sea bream has been performed previously in a few studies and in general, the results agree with our findings. For instance, the replacement of marine with vegetable ingredients, i.e., vegetal meal (VM) and/or VO, in the gilthead sea bream diet did not influence the overall alpha-diversity of gut bacteria in previous studies by Estruch et al. (2015), Parma et al. (2016) and Castro et al. (2019). However, unlike our results, almost 2-fold higher alpha-diversity values (e.g., No. OTUs, Shannon and Choa1) were found in the hindgut of gilthead sea bream in these previous studies (Estruch et al., 2015; Parma et al., 2016). This could be explained by the different age of fish used in the present study since there is evidence that alpha-diversity decreases with age in fish (Llewellyn et al., 2016; Heys et al., 2020). Rimoldi et al. (2018) found no differences in the alpha-diversity of gut bacteria in gilthead sea bream of similar size compared to the current study. No significant differences in richness and diversity of gut bacteria was found in the study of Piazzon et al. (2020) that fed a control or a plant-based diet to three groups of genetically selected gilthead sea bream families. Equally, dietary levels of FM, FO or their combination did not affect faeces and mucosa species richness or Shannon-Weaver diversity index in European sea bass (Torrecillas et al., 2017). Conversely, a recent analysis of gut microbial community in gilthead sea bream fed a diet with high VM and VO level has highlighted a significant increase of microbiota biodiversity compared to control fish fed a FM/FO based-diet (Parma et al., 2020). In the same fish species, Piazzon and colleagues (2017) reported instead that 58% and 84% of substitution of dietary FO by VO lowered autochthonous gut microbiota diversity leading to a dominance of the *Photobacterium* genus.

It is interesting to note the lower alpha-diversity found in the mucosa in comparison to the faeces samples. This agrees with previous studies in salmonid fish that have reported a higher biodiversity and richness of bacterial species hosted by intestinal lumen in comparison to the gut mucosa (Gajardo et al., 2016; Huyben et al., 2018; Rimoldi et al., 2019; Terova et al., 2019). In European sea bass, autochthonous microbiota was instead characterized by higher species richness than allochthonous (transient) microbiota (Rimoldi et al., 2020b).

Previous NGS studies on gilthead sea bream have found similar gut microbial profile dominated by Firmicutes and to a lesser extent by Proteobacteria, Actinobacteria and Bacteroidetes phyla, and by *Lactobacillus*, *Photobacterium*, *Corynebacterium* and *Propionibacterium* genera (Estruch et al., 2015; Parma et al., 2016; Parma et al., 2020). In the present study, Proteobacteria and Firmicutes were the dominant phyla in both, faeces and mucosa samples regardless of the diet, as previously reported in gilthead sea bream and other fish species (Estruch et al., 2015; Parma et al., 2016; Parma et al., 2020; Piazzon et al., 2017; Rimoldi et al., 2018; Rimoldi et al., 2019; Rimoldi et al., 2020a; Rimoldi et al., 2020b; Terova et al., 2019; Magalhães et al., 2020). Beta diversity analysis showed that neither resident nor transient intestinal communities were affected by dietary treatment. In contrast, a clear distinguished microbial profile was found in the faeces of gilthead sea bream fed a diet with a total replacement of FO with VO (Castro et al., 2019).

At the genus/family taxa level, LEfSe analysis revealed that fish fed 60% CO diet showed a higher abundance of *Corynebacterium* in the gut mucosa, indicating a possible association of this taxon with the 60% CO diet. The same bacterial genus increased in gut mucosa of slow-growth gilthead sea bream fed with a plant-based diet (Piazzon et al., 2020). This genus contributed to higher predictive pathways of fatty acid synthesis, since it, along with other genera belonging to Actinobacteria and Firmicutes phyla (e.g., *Lactobacillus*), is capable of producing short-chain fatty acids, such as acetate and butyrate, which act as an energy source and stimulate enterocytes proliferation in fish (Kihara & Sakata, 1997; Asaduzzaman et al., 2018). In European sea bass the genera *Clostridium*, *Corynebacterium* and *Staphylococcus* were only identified in fish fed with low FM/FO levels (Torrecillas et al., 2017). In the gut of Arctic charr, dietary marine-derived oil reduced viable counts of *Corynebacterium* in comparison to fish fed soybean and linseed oils (Ringo et al., 2002). In contrast, a study by Estruch et al. (2015) showed that *Corynebacterium* in the hindgut of gilthead sea bream remained the same when FM was replaced with VM. Therefore, either these results indicate that high inclusion of CO may change the mucosal environment that benefits the colonisation of *Corynebacterium*, or fish may select *Corynebacterium* to produce fatty acids to compensate for fish oil replacement in the diet. A study in Atlantic salmon, showed that most bacteria in the gut are transient with no evidence of adaptation to host environment (Heys et al., 2020), thus suggesting that *Corynebacterium* may have been selected by the host based on the demand for fatty acids.

Reduced weight gain in fish fed the 60% CO diet may be associated with a reduced abundance of *Lactobacillus* in the gut, which were instead enriched in fish fed control diet 0% CO. *Lactobacillus* is a lactic acid bacterium (LAB; Lactobacillales order) often used as a probiotic in aquafeeds and therefore, considered favourable due to its properties to stimulate and enhance gut development, digestive function, mucosal tolerance, immune response and disease resistance in fish (Merrifield et al., 2010; Ringøet al., 2018). Indeed, a higher Firmicutes:Proteobacteria ratio is generally regarded as beneficial because it is positively correlated to the abundance of LAB. The high abundance of Firmicutes (32–68%) found in the gut agrees with most of the previous studies on gilthead sea bream (Estruch et al., 2015; Parma et al., 2016; Rimoldi et al., 2018). Only Piazzon et al. (2017) found a low percentage of Firmicutes (1–28%) in the gut of gilthead sea bream and these authors suggested Firmicutes may be more transient than the other phyla.

The predictive function of the gut microbiome of fish has only been investigated in a small handful of studies (Lyons et al., 2017; Wei et al., 2018; Yang et al., 2019; Piazzon et al., 2019; Qiao et al., 2020; Piazzon et al., 2020) and to our knowledge this is one of the first investigations of gilthead sea bream. The absence of significant effects of diet or gut section indicate that replacing FO with CO did not drastically alter the composition and function of microbes in the gut. It also reveals that the function of allochthonous (transient) and autochthonous (resident) bacteria are similar and analysing each gut section separately may not be necessary.

In regards to metabolic pathways, the predictive functions of gut microbes in the present studies agree with previous findings that they are mainly associated with the metabolism of carbohydrates, amino acids, energy and to a lesser extent lipids (Liu et al., 2016; Dehler, Secombes & Martin, 2017; Lyons et al., 2017; Wei et al., 2018; Yildirimer & Brown, 2018). Actinobacteria, such as *Corynebacterium* and *Gordonia* (Fig. 7), dominated the pathway of fatty acid synthase, which have low abundances in the gut but may be key contributors to lipid metabolism. In Arctic charr, feeding VOs increased viable counts of *Corynebacterium* in comparison to fish fed marine-derived oils (Ringo et al., 2002). A similar result was found in the present study especially with regard to the mucosa of fish fed with 60% CO diet. High VO diets may increase the abundance of fatty acid producing bacteria to compensate for the lack of fatty acids in the diet. Piazzon et al. (2020) found striking changes in the metabolic capacity of intestinal bacteria of gilthead sea bream in response to plant-based diets. In particular, 59, 84 and 15 pathways were found to be changed in fast growth, intermediate growth, and slow growth sea bream families, respectively. Among them, pathways related to infection, inflammation or immune activation, were the most influenced by diet and genetic selection. However, it should be noted that these results are only functional predictions and the deep shotgun sequencing of all bacterial genomes is preferred (Langille et al., 2013). Indeed, recently Sun, Jones & Fodor (2020) reported that the utility of PICRUSt is likely limited outside of human samples and that the development of tools for gene prediction specific to different non-human samples is needed. However, it is also true that the use of this meta-genomic tool represents the only available strategy to determine metabolic capacities of non-human microbiomes. For this reason, we only focused on the metabolic pathways instead of the disease related pathways. Functional metagenomics data should be validated or complemented with empirical approaches, such as sequencing the bacterial transcriptome, to give a more detailed description of metabolic pathways that are actually active in the gut microbial community.

## Conclusions

This study demonstrated that diets can replace up to 40% of fish oil with CO without negative effects on growth performance, fillet fatty acid profile and gut microbiome of gilthead sea bream. Final weight and fillet EPA were significantly reduced in fish fed the 60% CO diet. Alpha and beta-diversities of gut bacteria both in the faeces and mucosa were not affected by any dietary treatment, although a few indicator bacteria, such as *Corynebacterium* and *Rhodospirillales*, were associated with the 60% CO diet. This study highlighted the difference in alpha-diversity between sample type, which was highest in the feeds and lowest in the gut mucosa. Reduced growth of fish fed 60% CO diet may be due to the lack of EPA and DHA in the CO and a reduction in lactic acid bacteria, specifically *Lactobacillus*, in the feaces and gut mucosa. Feed conversion was not affected by CO inclusion, thus reduced growth was not due to reduced digestibility but rather driven by nutrient demand, e.g., n-3 PUFA. Metagenomic analysis revealed similar predictive function of the gut microbes, although a higher abundance of *Corynebacterium* in the mucosa of fish fed 60% CO diet increased the KEGG pathway of fatty acid synthesis and may act to compensate for the lack of fatty acids in the diet. These results indicate that 40% of FO can be replaced with CO in diets for gilthead sea bream and demonstrates that CO can be an alternative oil ingredient in aquafeeds.

##  Supplemental Information

10.7717/peerj.10430/supp-1 Supplemental Information 1 Fish growth data.

10.7717/peerj.10430/supp-2 Supplemental Information 2 Raw data for lipid analysis.

## Additional Information and Declarations

### DNA Availability

The following information was supplied regarding the deposition of DNA sequences: The raw 16S rRNA sequence reads are available in the Sequence Read Archive: PRJNA657669.

### Data Deposition

The following information was supplied regarding data availability: The raw data are available as Supplementary Files.
